# The Doctor of Physical Therapy Admission Test (DPTAT): A Vision for a Primary Care, Doctoring Profession

**DOI:** 10.1007/s40670-025-02523-4

**Published:** 2025-09-30

**Authors:** Nathan J. Savage

**Affiliations:** Department of Physical Therapy, Winston-Salem State University, 601 S. Martin Luther King Jr. Drive, 336 F.L. Atkins Building, Winston-Salem, NC 27110 USA

**Keywords:** Admissions, Doctoral education, National Physical Therapy Examination, Predictive validity, Primary care

## Abstract

The Doctor of Physical Therapy Admission Test (DPTAT) is a profession-specific instrument being proposed to address the limitations of traditional admissions metrics for predicting DPT program performance and national licensure examination success. By aligning with key cognitive, affective, and psychomotor demands of contemporary physical therapist education and clinical practice, including the emergence as primary care providers, the DPTAT seeks to identify candidates most likely to succeed in doctoral education and enter the profession. This *Commentary* outlines the theoretical rationale and validation strategies of the proposed DPTAT within the broader context of medical education and the demands of modern healthcare systems.

## Contemporary Physical Therapist Education and Clinical Practice

The physical therapy profession is at a critical inflection point where the vision of autonomous, first-contact, primary care physical therapist practice is increasingly becoming a reality [1–9]. As such, professional Doctor of Physical Therapy (DPT) program curricula must continue to evolve by selecting candidates capable of meeting the emerging needs of contemporary physical therapist education and clinical practice. DPT education in the United States is moving toward competency-based models, which will facilitate the training of advanced practice and primary care providers by including content and experiences focused on clinical reasoning, differential diagnosis, and interprofessional communication and collaboration [10, 11]. However, admissions practices among professional DPT programs have failed to evolve in parallel with other doctoral-level primary care professions (e.g., medicine, dentistry, optometry, etc.) to improve the method of selecting appropriately qualified candidates through profession-specific metrics [12–14].

No standardized entrance examination currently exists for physical therapy education. In fact, the author could find no evidence that the American Physical Therapy Association or any other physical therapy-related professional organization has ever proposed or advocated for a profession-specific entrance examination. DPT programs continue to rely on a constellation of legacy metrics to select candidates, including undergraduate grade point average (GPA), graduate record examination (GRE) scores, essays, personal statements, and subjective interviews [15–17]. These metrics—whether considered individually or in combination—have performed unevenly and generally poorly for predicting student success in DPT programs and success on the National Physical Therapy Examination (NPTE) [18–26]. Furthermore, the existing cognitive and noncognitive metrics fail to fully capture the multifaceted competencies that define excellence in contemporary DPT education and clinical practice.

This *Commentary* proposes the development and validation of the Doctor of Physical Therapy Admission Test (DPTAT), a conceptual tool that can strengthen existing admissions practices by providing robust predictive validity for the selection of candidates most likely to succeed in their DPT education and successfully pass the NPTE on their first attempt. The DPTAT would be the first standardized, evidence-based, and profession-specific evaluative tool used to bolster contemporary DPT education and clinical practice to meet the emerging needs of a primary care, doctoring profession.

This following is an outline of the theoretical underpinnings and practical application of the DPTAT based on empirical evidence and modeled after the successes (and failures) of admission tests in other primary care professions [12, 13]. The vision of the DPTAT represents a philosophical pivot for the physical therapy profession, reframing readiness for contemporary DPT education, seeking to identify candidates who can *think*, *reason*, *relate*, and *adapt* to the complex realities of modern healthcare systems. The DPTAT aims to establish a new paradigm in physical therapist education—rigorous, theory-informed, evidence-based, accessible, and profession-defining.

## The Status Quo Falls Short

While considerable variation exists in admissions criteria, most DPT programs consider a mixture of cognitive (e.g., GPA and GRE scores) and noncognitive (e.g., interviews, essays, letters of recommendation, and clinical experiences) factors [27–29]. The GRE evaluates general cognitive skills with sections on verbal reasoning, quantitative reasoning, and analytical writing, but does not measure discipline-specific knowledge and lacks contextual relevance for allied health professions [30, 31]. While undergraduate GPA and GRE scores have been shown to have some predictive validity for student success in DPT education and success on the NPTE, some authors have questioned the value of these legacy metrics, suggesting that they are limited and may be misleading among certain applicants [18–24, 29].

Despite these criticisms, a recent systematic review of 31 studies found that the inclusion of cognitive outcomes, namely GRE scores, undergraduate cumulative GPA, and undergraduate pre-requisite GPA, provides evidence of which students are most likely to manage the rigor of DPT education and successfully pass the NPTE [27]. Alternatively, another systematic review by the same author evaluated 29 studies of programs using holistic admissions practices and found that noncognitive outcomes were insignificant or inconsistently associated with student DPT program success and NPTE performance [28]. These findings are not surprising given the subjective nature of personal statements, written essays, and interviews [32]. The authors found some evidence that personal attributes like emotional intelligence and grit may predict student success; however, they also found that sociodemographic variables were more likely to predict poorer academic and test performance, namely older age, individuals identifying as an underrepresented racial/ethnic groups, and individuals reporting English as a second language [28].

Current admissions practices may fail to adequately evaluate other desirable and possibly essential attributes and characteristics of candidates seeking to enter contemporary DPT education and clinical practice, including as primary care providers. The DPTAT is proposed as a tool that would be designed to identify candidates with critical reasoning fluency, probabilistic thinking, proprioceptive-spatial modeling, and applied biomechanical reasoning—capacities fundamental to differential diagnosis, movement analysis, and autonomous, first-contact, primary care settings [10, 11]. Additionally, the DPTAT will emphasize professional dispositions such as adaptability under uncertainty, cognitive empathy, and ethical judgment within the context of interprofessional collaboration and complex decision-making. These attributes, rooted in the cognitive and affective demands of contemporary physical therapist education and clinical practice, seek to offer a more valid and profession-defining approach to DPT program admissions.

Considering the tremendous investment of time, effort, and financial resources required to obtain a DPT degree and enter the profession, program admissions practices should embrace the solemn responsibility of being student-centered, evidence-based, and committed to selecting candidates that are most likely to succeed. Alternatively, when admissions practices are not informed by the available data, students and programs are put at an increased risk of setbacks and failures, including higher rates of academic difficulty and attrition, delayed clinical readiness, lower graduation rates, and declining NPTE passage rates [15, 16, 22, 33–35]. The consequences of ill-informed or misguided admissions practices have real-world effects on students’ psychological and financial well-being, including the devastating effects of failure to complete a professional doctoral degree after having invested significant time and money and not reaping the benefits of earning potential as an early career provider [15, 17, 27, 28, 33–36]. Additionally, student attrition and declining NPTE passage rates reflect poorly on institutions, which can negatively impact their ability to recruit top candidates who are increasingly drawn to other professions.

The selection of appropriate cognitive and noncognitive factors to be used in DPT program admissions should be evidence-based and remain focused on the selection of candidates most likely to graduate and enter the profession [21, 22, 29, 32]. The consequences of misalignment between program demands and candidate preparation are more than just academic: students ill-equipped for the rigors of doctoral education are more likely to experience psychological distress, academic difficulty, attrition, or program dismissal [19, 33–37]. Entrance examinations have been shown to inform student risk of failure or academic difficulty in graduate health professions education, including medicine, nursing, and public health [36, 38–42]. A recent program-specific study found that a prerequisite entrance examination can improve predictive validity for students’ first-semester DPT program GPA. While the findings in this study were modest, explaining 24% of the shared variance, the implications are profound and support the theoretical rationale for developing discipline-specific cognitive metrics [33]. These data signify a challenge and an opportunity: given the increasing focus on value-based education and clinical practice, can the physical therapy profession develop a tool to support selection of candidates that is rigorous and standardized, predictive but not reductionist, and that provides equal opportunity without sacrificing precision and relevance?

## Framework: Proposed Development and Validation of the DPTAT

The vision of the DPTAT is an evaluative tool that reflects the unique cognitive, affective, and psychomotor demands of contemporary physical therapist education and clinical practice. The DPTAT will depart from generic aptitude tests and instead operationalize a profession-specific instrument that evaluates student readiness through construct-aligned domains, each rooted in a theoretical foundation and supported by empirically validated competencies (Table 1). This multi-domain framework is intended to ensure that a proposed DPTAT would not just be a proxy for DPT program success and a valid predictor of NPTE success but could represent professional excellence that considers physical therapists as primary care professionals that can meet the emerging needs of modern healthcare systems.

**Table 1:** Structure, competency targets, and rationale for the Doctor of Physical Therapy Admission Test (DPTAT)

DPTAT Section	Competency	Construct	Questions	Theoretical Rationale
Foundational Sciences	Biomedical knowledge integration	Scientific knowledge of human structure, movement, & function	50 multiple-choice	Assesses prerequisite knowledge foundational to clinical examination, clinical safety, & academic success
Problem-Solving & Critical Thinking	Clinical reasoning & differential diagnosis	Logical & analytical reasoning	25 logic puzzles & clinical vignettes	Evaluates flexible thinking, diagnostic acumen, & decision-making under clinical uncertainty
Spatial Awareness & Movement Analysis	Body-system spatial reasoning & movement analysis	2D/3D visual-spatial processing & kinesthetic cognition	25 images & videos focused on spatial orientation tasks	Measures perceptual-motor skills central to physical examination, movement analysis, & interventions
Situational Judgment	Resilience, ethics, & adaptability in patient care	Ethical reasoning, empathy, & professional adaptability	20 rank-ordered responses to interpersonal scenarios	Aligns with affective domain competencies; Predicts professional conduct & interpersonal effectiveness
Written & Verbal Communication	Empathic & professional communication	Clarity, empathy, & professional tone	5 structured written & audio responses	Captures articulation of clinical reasoning & patient-centered communication essential for care delivery

The following summarizes envisioned domains for the proposed DPTAT:

- **Foundational Sciences**. This domain will evaluate biomedical knowledge essential for integrating anatomy, kinesiology, biomechanics, exercise physiology, and pathophysiology in a clinical reasoning framework. Representative of DPT program pre-requisite coursework emphasizing movement science.
- **Problem-Solving and Critical Thinking**. This domain will evaluate inductive and deductive reasoning, pattern recognition, and flexible integration of information. Draws on clinical reasoning literature (e.g., hypothetico-deductive models, dual-process theory).
- **Spatial Awareness and Movement Analysis**. This domain will evaluate visual-spatial reasoning, mental rotation, kinesthetic empathy, and anatomical orientation. Grounded in spatial intelligence theory and embodied cognition, capturing essential skills for physical examination, manual therapy, movement analysis, and diagnostic imaging.
- **Situational Judgment**. This domain will evaluate problem solving, ethical reasoning, professionalism, interpersonal adaptability, and reflective judgment. Leverages professionalism frameworks (e.g., American Physical Therapy Association’s Core Values), virtue ethics, and Korthagen’s model of reflection (i.e., action, looking back, awareness, alternatives, and trial).
- **Written and Verbal Communication**. This domain will evaluate clarity of thought, patient education capability, and argumentation in both written and spoken form. Integrates rhetorical theory and narrative healthcare concepts to assess the clarity, empathy, and relational acuity required for patient-centered care.

**Fig. 1:**
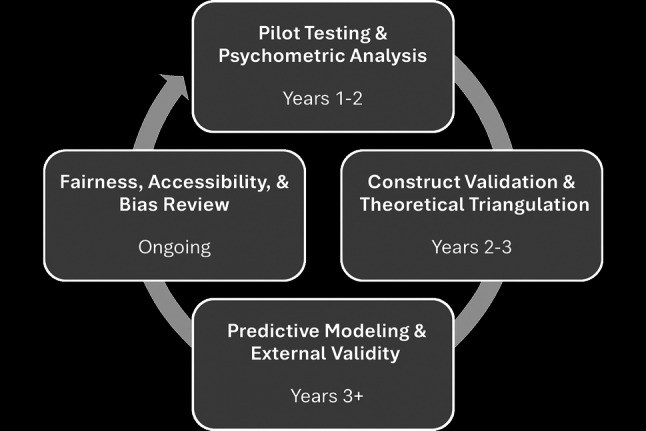
Proposed process of development and validation of the Doctor of Physical Therapy Admission Test (DPTAT)

Any future development and validation of the DPTAT would need to be guided by contemporary standards of educational measurement and an argument-based approach to validation [43, 44]. The proposed validation model (Fig. 1) ensures psychometric robustness and contextual relevance. While an overview of the processes and estimated timeframes is provided as a suggested framework, the development and validation of the proposed DPTAT are unlikely to occur in a linear fashion; it will likely involve iterative steps not currently identified, and the process is likely to be refined based on the results of pilot testing to strengthen the tests’ predictive validity.

In the development of the proposed DPTAT, pilot testing and data collection will be used to inform adjustments to examination content. For example, items would undergo rigorous analysis for difficulty, discrimination, and reliability across a diverse sample of DPT students and DPT program applicants. Next, expert panels will review the validity of domain alignments, and factor analyses will assess dimensional integrity to ensure that domains are linked to existing theoretical frameworks, ensuring that the DPTAT is more than a knowledge inventory but a reflection of the profession’s epistemology. Finally, longitudinal studies will need to be conducted to evaluate the predictive validity of the DPTAT for student outcomes. Multivariate regression analyses can measure associations between DPTAT scores and key outcomes like DPT program GPA, DPT program comprehensive assessments, clinical competencies, and NPTE passage rates. Once an instrument is established, ongoing evaluation for fairness, accessibility, and bias will be conducted using differential item functioning analysis to identify group-level bias across demographic variables. Design features such as screen-reader compatibility, extended time, and plain-language guidelines will help ensure access for all test takers, including neurodiverse and multilingual candidates.

While the DPTAT is being designed to improve the selection of candidates most prepared to meet the rigors of doctoral training and successfully pass the NPTE, emphasis will also be placed on providing equal opportunity, rewarding predictive merit, and being accessible and affordable for DPT program applicants. The vision of the DPTAT is one that avoids over-reliance on costly preparatory materials and requirements for non-predictive metrics. A future DPTAT could leverage artificial intelligence for grading and structured rubric-based scoring to mitigate bias in the evaluation of the open-ended, free-response sections. A proposed DPTAT would include a commitment to accessibility, with potential accommodations for neurodiverse candidates and non-native English speakers through format innovations including real-world and clinically based scenario items, multi-modal response formats (e.g., image-based, video-based, verbal-based free-response items). These innovations also increase the probability of evaluating candidate attributes necessary for academic success and clinical competence, ensuring that the DPTAT is not merely a filter, but a fair and faithful reflection of contemporary physical therapist education and clinical practice.

## DPTAT vs Other Profession-Specific Entrance Examinations

The development of the DPTAT draws both inspiration and caution from long-standing profession-specific admission instruments. The MCAT® and DAT, entrance examinations for medicine and dentistry respectively, offer complementary models of competency assessment in health professions (Table 2). These tools have demonstrated utility in standardizing selection criteria, elevating academic expectations, and creating shared thresholds for entry into professional medical and dental education programs [12, 13]. For most medical and dental programs, these entrance examinations are used in addition to other cognitive and noncognitive variables to help predict student success.

**Table 2:** Proposed Doctor of Physical Therapy Admission Test (DPTAT) sections mapped to the Medical College Admission Test (MCAT®) and the Dental Admission Test (DAT) influences

DPTAT Section	Purpose	Influence	Example Tasks
Foundational Sciences	Foundational knowledge in anatomy, biomechanics, exercise physiology, kinesiology, & neurophysiology	• DAT natural sciences • MCAT® biological & biochemical foundations of living systems	Identify planes of motion; Evaluate muscular compensations related to movement; Interpret a dermatomal map
Problem-Solving & Critical Thinking	Assess spatial cognition relevant to human movement and posture	• DAT Perceptual Ability Test	Visualize joint axis in multiple planes; Mental rotation of anatomical segments
Spatial Awareness & Movement Analysis	Evaluate hypothesis-driven reasoning in clinical contexts	• MCAT® critical analysis & reasoning; psychological, social, & biological foundations of behavior	Select appropriate test or intervention; Detect red & yellow flags in a case vignette
Situational Judgment	Assess understanding of statistical concepts & study design	• DAT quantitative reasoning • MCAT® scientific reasoning	Interpret a forest plot; Apply diagnostic specificity; Identify bias based on study design
Written & Verbal Communication	Appraise & extract clinical-relevant information from literature	• DAT reading comprehension • MCAT® critical analysis & reasoning	Interpret information from a Clinical Practice Guideline; Evaluate patient-reported outcomes data

The MCAT®, developed by the Association of American Medical Colleges, emphasizes interdisciplinary integration and critical reasoning across four sections: biochemical and physical sciences, verbal analysis (i.e., critical analysis and reasoning skills), biological systems, and psychosocial foundations. The MCAT® blends foundational knowledge with passage-based interpretation, modeling the kind of contextual problem-solving and ethical reasoning demanded in modern medicine. The MCAT® is structured to prioritize both scientific fluency and humanistic literacy, offering a construct-valid predictor of academic and clinical performance. In contrast, the DAT, which is administered by the American Dental Association, leans toward breadth of factual scientific knowledge, perceptual-spatial aptitude (i.e., Perceptual Ability Test section), and applied quantitative reasoning. The DAT reflects the manual-visual demands of dental training while maintaining emphasis on core biology and chemistry. While less integrative than the MCAT®, the DAT is designed to underscore the value of a profession-specific aptitude test (Table 3).

**Table 3:** Comparison of the proposed Doctor of Physical Therapy Admission Test (DPTAT) with the Medical College Admission Test (MCAT®) and Dental Admission Test (DAT)

Feature	MCAT®	DAT	DPTAT
Profession	Medicine	Dentistry	Physical Therapy
Administering Body	Association of American Medical Colleges	American Dental Association	TBD
Cognitive & Affective Domains	Biology, chemistry, physics, psychology, sociology, & critical reasoning	Natural sciences, perceptual ability, reading comprehension, & quantitative reasoning	Foundational sciences, problem-solving, & communication
Psychomotor Domains	None	Perceptual Ability Test (2- & 3-dimensional spatial reasoning)	Spatial awareness, movement analysis, & mental rotation
Situational Judgment/Ethics	Situational Judgment Test (pilot)	Not included	Situational judgment (aligned with APTA Core Values & ethical reasoning frameworks)
Communication Assessment	Reading comprehension	Reading comprehension	Argumentation, empathy, verbal clarity, & clinical communication
Delivery Mode	Computer-based, standardized	Computer-based, standardized	Computer-based, standardized, & multimodal
Cost & Preparation	High; test prep industry dependent	High; test prep industry dependent	Low; open-access preparation materials
Predictive Validity	Extensive evidence	Moderate evidence	TBD

*APTA* American Physical Therapy Association

Together, these profession-specific entrance examinations provide a framework with fertile ground for developing the vision of the DPTAT—one that blends anatomic and physiological depth, movement science, clinical reasoning, evidence-based decision-making, and interprofessional communication. From biomechanics to behavior, such an evaluative tool could mirror the demands of contemporary physical therapist education and clinical practice while honoring the cognitive, affective, and psychometric rigor found in these medical and dental counterparts.

The envisioned DPTAT is an instrument intended to share similarities with the MCAT® and DAT, namely a purpose-built design (i.e., assessing competencies aligned with respective profession), construct-based domains (i.e., foundational knowledge and higher-order cognitive processing like critical thinking and scientific reasoning), and predictive orientation (i.e., forecasting readiness and performance in professional education and licensure). However, critical differences emerge when comparing the DPTAT’s design philosophy and content domains with its counterparts:

- **Psychomotor and Spatial Reasoning Emphasis**. Unlike the MCAT® or DAT, which primarily assess cognitive and verbal reasoning skills, the DPTAT will include spatial awareness and embodied cognition components, reflecting the physical therapist’s need for visual-spatial interpretation, movement analysis, and hands-on diagnostic acumen.
- **Professional Identity and Affective Reasoning**. The DPTAT will incorporate a situational judgment domain grounded in professional values, ethics, and adaptive behavior, more closely resembling recent innovations like the Association of American Medical College’s *Situational Judgment Test* but designed from inception to reflect the American Physical Therapy’s Core Values and interpersonal demands of the therapeutic alliance between patient and provider.
- **Communication Beyond Scientific Writing**. Whereas the MCAT® and DAT emphasize formal scientific reading and reasoning, the DPTAT will include rhetorically informed communication assessments—evaluating narrative clarity, patient education ability, and empathy-laden language as essential dimensions of care delivery in contemporary physical therapist education and clinical practice.
- **Accessibility and Format Innovation**. The DPTAT will be designed with delivery methods to reach all candidates (e.g., scenario-based video prompts, artificial intelligence-supported rubric scoring, open access preparation materials, etc.) to reduce the need for expensive test preparation materials, improve accessibility for diverse learners, and reflect multimodal professional tasks—departing from the heavily text-based and time-based pressure of the MCAT® and DAT.

## Conclusion: From Knowledge to Wisdom

The vision of developing and validating the DPTAT is more than merely curricular reform; rather it represents a paradigm shift and cultural declaration as physical therapy continues to evolve as a primary care, doctoring profession. By investing in a theory-informed, psychometrically sound admissions instrument, contemporary physical therapist education demonstrates maturity and alignment with other doctorate-level, primary care health professions. The vision of the DPTAT also aligns with value-based care priorities, selecting candidates based on traits like adaptability, ethical reflexivity, and interprofessional communication—attributes increasingly linked to improved patient outcomes and satisfaction [45]. Like the long-standing experience of medical and dental programs, entrance testing should be viewed as an adjunctive measure to strengthen the selection of candidates most likely to succeed in their education and enter the profession, not as the only measure or even the most important measure of benchmarking later student success. Additionally, potential positive downstream effects include those for candidates (i.e., more meaningful, transparent, and profession-relevant experience when seeking entrance into a professional DPT program), for DPT programs (i.e., strengthens admissions metrics by providing an evaluative tool with predictive validity to identify candidates with a high potential for success), and for the physical therapy profession (i.e., enhanced legitimacy in policy discussions about scope of practice, reimbursement, and interprofessional status while evolving as a primary care profession).

Rather than serve as a gatekeeper for academic elitism, the DPTAT aspires to become a selector of professional promises: of those who can reason clinically, connect interpersonally, adapt ethically, and lead within complex health systems. This divergence from legacy metrics is not a detour but an evolution—aligned with the forward-thinking identity of the physical therapy profession as it enters its second century. Physical therapy, too, is a profession lived in moments of clinical connection, reflective decision-making, and embodied presence not in raw test scores. The DPTAT aims to honor that ethos not by abandoning measurement, but by elevating it. By capturing the full spectrum of readiness, namely cognitive, affective, and psychomotor, the DPTAT can offer a more just, accurate, and profession-specific lens through which to view the clinicians of tomorrow. This is not simply an evolution of testing; rather, it is an articulation of professional identity, a declaration of values, and a call to action. Beginning the process of developing and validating an instrument like the DPTAT, physical therapy takes its rightful place among the learned doctoring professions as innovators. 

signaling that physical therapists have both the intellectual rigor and professional accountability to legitimize our profession’s claim to autonomous, advanced practice primary care roles.

The DPTAT seeks to identify candidates not merely for their academic potential, but for their alignment with the profession’s evolving identity: diagnostically astute, ethically grounded, and interpersonally effective. This transformation is needed to strengthen the physical therapy profession’s political and economic standing by aligning admission standards with those of other recognized primary care professions [12–14]. By aligning admissions metrics with the clinical and professional demands of contemporary physical therapist practice, making the vision of the DPTAT a reality will redefine what readiness looks like and ensure that our professional education cultivates clinicians with knowledge and wisdom in the service of others.
